# Bacterial restriction-modification systems: mechanisms of defense against phage infection

**DOI:** 10.52601/bpr.2025.240070

**Published:** 2025-10-31

**Authors:** Haoyang Kang, Ang Gao, Yalan Zhu

**Affiliations:** 1 School of Life Science, Beijing Institute of Technology, Beijing 100086, China

**Keywords:** R-M system, Bacteriophage, DNA Restriction, DNA Modification, Anti-restriction

## Abstract

Bacteria have evolved various defense mechanisms against bacteriophage invasion, driven by a long-term evolutionary arms race. Among them, restriction-modification (R-M) systems, which protect bacteria by DNA restriction and modification, are prominent. R-M systems are categorized based on their DNA methylation mechanisms, restriction activities, and cofactor dependencies. Here, we review the molecular basis of R-M systems and the diverse strategies used by bacteriophages to evade these defenses. Furthermore, we highlight the co-evolutionary dynamics between R-M systems and phage countermeasures, providing insights into new approaches to combat bacterial resistance and pathogenicity. Beyond their defensive roles, the multifunctionality of R-M systems in broader biological and biotechnological contexts is also discussed.

## INTRODUCTION

Phages, one of the most abundant organisms on the Earth, thrive by infecting bacteria, establishing a classic parasite-host dynamic. Over evolutionary time, bacteria have evolved a variety of sophisticated defense mechanisms to counter phage infection, driven by an ongoing arms race with these parasites. These mechanisms include adsorption resistance (Sanders and Klaenhammer 1983), the restriction-modification (R-M) system (Obarska *et al.* 2006), CRISPR-Cas system (Jansen *et al.* 2002), abortive infection (Tran *et al.* 1999), and recently discovered systems such as BREX (Goldfarb *et al.* 2015), DISARM (Ofir *et al.* 2018), pAgos (Swarts *et al.* 2014), Thoeris (Ka *et al.* 2020), Gabija (Cheng *et al.* 2021), Zorya, Druantia (Doron *et al.* 2018), DRT2 (Wilkinson *et al.* 2024), AVAST (Gao *et al.* 2020), and CARD domains mediate antiphage system (Wein *et al.* 2025).

Among these, the R-M system is a widely distributed innate immune mechanism (Huo *et al.* 2019) found in over 90% of bacterial genomes (Blow *et al.* 2016; Roberts *et al.* 2010). It plays a central role in defending bacteria against bacteriophages and foreign DNA elements (Adamczyk-Poplawska *et al.* 2011) through enzymatic activities. The R-M system consists of two main types of enzymes: modification enzymes (MTases), which methylate DNA by transferring methyl groups to specific adenine or cytosine residues, and restriction enzymes (REases), which cleave DNA at recognition sites (Sitaraman 2016; Teklemariam *et al.* 2023). The genes encoding these proteins are found on bacterial chromosomes, resistance transfer factor plasmids, and phage template chromosomes (Arber 1965b; Luria and Human 1952; Watanabe *et al.* 1966).

Awareness of restriction and modification grew in the early 1950s, when microbiologists observed that the bacterial strain of the last host in which phages replicated influenced the host range of those phages (Luria and Human 1952). This phenomenon, called host-controlled modification (Arber 1965a), was initially thought to merely mark DNA for host recognition. However, subsequent studies have revealed that these modifications protect DNA from cleavage by restriction enzymes (Walder *et al.* 1983).

DNA methylation, the hallmark of the R-M systems, occurs either selectively on one or both strands of host DNA, safeguarding newly replicated DNA from enzymatic cleavage. The most common modification is *N*^6^-methyladenine (m^6^A), found in both prokaryotes and eukaryotes (Hattman *et al.* 1978; Vanyushin *et al.* 1968), which is essential for regulating gene expression and controlling virulence through phase on-off expression (Seib *et al.* 2020). Another modification, *N*^4^-methylcytosine (m^4^C), was previously thought to be unique to bacteria, but has recently been identified as an epigenetic mark in eukaryotic genomes (Rodriguez *et al.* 2022). Notably, all Type I, II, and III R-M systems can produce m^4^C. In addition, *C*^5^-methylcytosine (m^5^C), prevalent in both bacteria and eukaryotes, serves as the primary methylated base in eukaryotes (Hattman *et al.* 1978).

Cooperation between MTases and REases in R-M systems follows two distinct patterns. In the first, REases cleave DNA sequences that are not modified by their cognate-modifying MTases (Morgan *et al.* 2016). In the second, MTases modify target sequences that are then degraded by REases (Stewart *et al.* 2000). R-M systems are classified into five types based on their components and mechanisms. Type I, II and III R-M systems typically follow the first cooperation pattern, targeting unmethylated DNA for cleavage. In contrast, Type IV R-M systems exhibit reduced specificity, cleaving methylated DNA instead of unmethylated sequences (Roberts *et al.* 2003). Another variant, the DNA phosphorothioation (PT) R-M system, uses phosphorothioation instead of methylation to modify DNA sequences, providing a unique defense strategy (Wang *et al.* 2007).

The evolution of bacterial defense systems is closely related to the evolution of phage countermeasures. The prolonged arms race between bacteria and phages has driven the development of diverse protective mechanisms, allowing both to adapt and survive each other's ‘attacks’. It’s a coevolution process that both of them achieve reciprocal adaptation and counter-adaptation.

This review comprehensively summarizes advances in the classification of various R-M systems, focusing on their recognition sequences, cleavage sites, cofactors, and structures (Table 1). It also explores their roles beyond defense against bacteriophage infection, such as their impact on gene expression and phase variation (DebRoy *et al.* 2021). For example, some R-M systems act as selfish mobile genetic elements capable of maintaining themselves within a host genome (Naito *et al.* 1995). The extensive and versatile Type II endonuclease family has significant applications in genetic engineering (Tóth *et al.* 2014). In addition, R-M systems are involved in the regulation of horizontal gene transfer in bacteria (Chen *et al.* 2021; Huo *et al.* 2019; Kennaway *et al.* 2012), a process that is critical for the spread of antibiotic resistance genes. Understanding the mechanism of regulation of the R-M system could provide critical insights into combating the global challenge of antibiotic resistance, as horizontal gene transfer is a primary mechanism for disseminating resistance genes (Ventola 2015). Phage therapy, which made some progress when used to treat drug-resistant Mycobacterial disease (Dedrick *et al.* 2023), demonstrates the potential application of phages in combating antibiotic resistance (Kumaran *et al.* 2018; Tanji *et al.* 2005). Furthermore, this review also explores bacterial defense mechanisms and the phage strategies for evading the R-M system, providing a comprehensive overview of the ongoing co-evolutionary dynamics. By consolidating these findings, the review aims to facilitate future research that delves deeper into the molecular intricacies of the R-M system.

**Table 1:** Characteristics of different types of R-M systems

	Type I	Type II	Type III	Type IV	PT
Take as an example	*Eco*R124I	*Bam*HI	*Eco*P15I	McrBC	Dnd system
Common components	HsdS, HsdM, HsdR	REase, MTase	Res, Mod	McrBL, McrBS, McrC	DndABCDE for modification DndFGH for restriction
	MTase: M_2_S_1_		MTase: Mod_2_		
	REase: R_2_M_2_S_1_		REase: Res_1_Mod_2_		
Restriction cofactors	ATP, Mg^2+^, AdoMet	Mg^2+^, AdoMet	ATP, Mg^2+^, (AdoMet)	GTP, Mg^2+^	ATP, (Mg^2+^)
Modification cofactors	AdoMet	AdoMet	AdoMet	−	ATP, L-cysteine
Recognition sequences	Asymmetric separate DNA sequences 5’-GAAN_6_RTCG-3’	4−8 bp palindromic DNA sequences 5’-GGATCC-3’	5−6 bp non-palindromic sequences 5’-CAGCAG-3’	R^m^C(N_40-80_)R^m^C^*a*^	Damage DNA in a non-sequence-specific manner
Cleavage sites	>1 kb away from the recognition sequences		~25 bp away on the 3’ side of the recognition sequences	Two recognition sites separated by 40 to 4000 bp	
^*a*^ R^m^C means DNA containing 5-hydroxymethylcytosine, 5-methylcytosine or 4-methylcytosine preceded by a purine residue					

## MECHANISMS OF R-M SYSTEMS

### Type I R-M system

**Figure 1:**
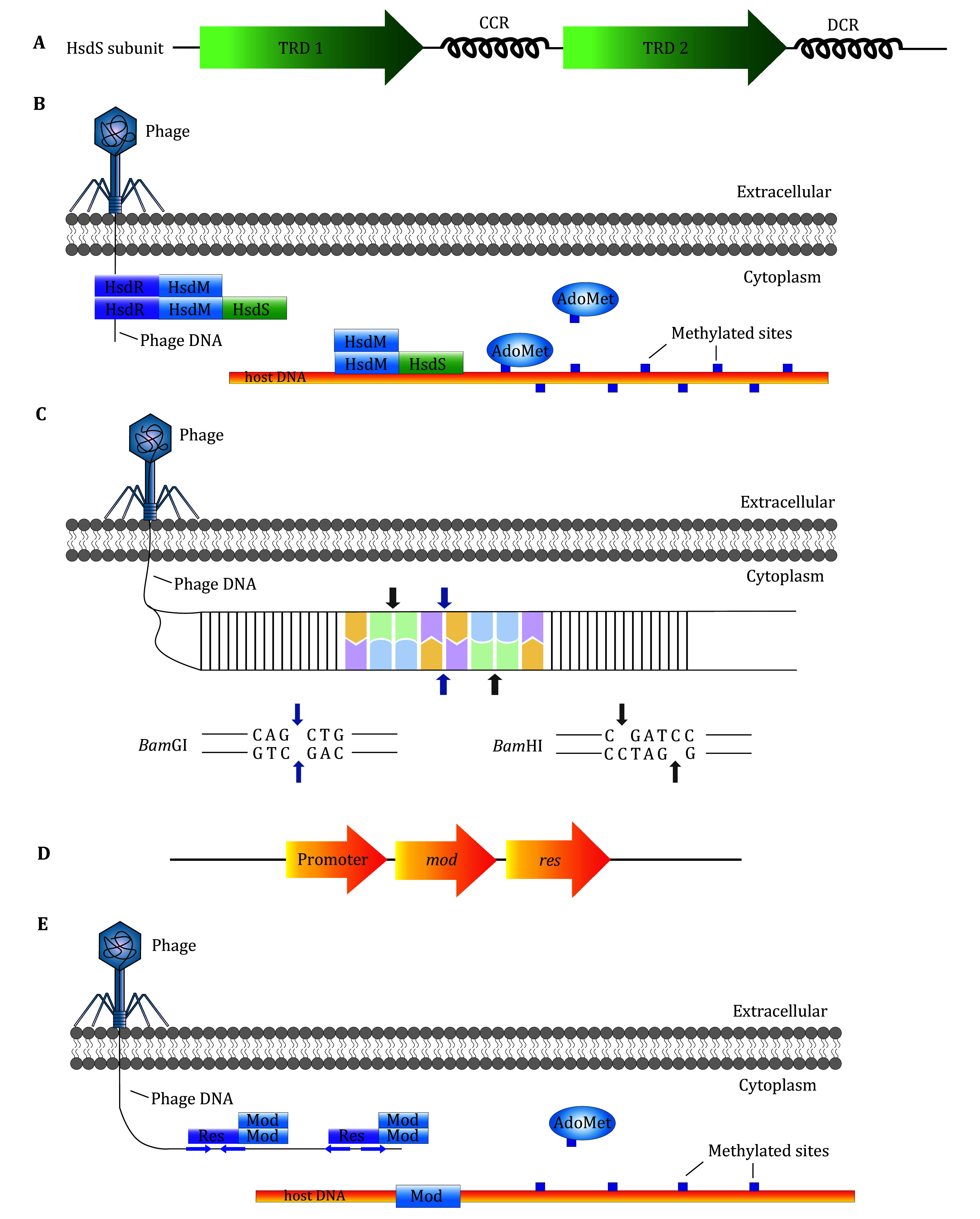
Mechanisms of Type I-III R-M Systems to cleave exotic phage DNA. **A** The two TRDs of the HsdS subunit are separated by central CR (CCR). Distal CR (DCR) is attached to the C terminus. Curves stimulate the CRs’ structure. **B** The R_2_M_2_S_1_ complex performs the restriction function of Type I R-M system while the M_2_S_1_ complex methylates the host DNA. AdoMet acts as a methyl donor in Type I R-M system. **C** Orthodox Type II R-M system recognizes a 4-8 bp palindrome sequence. Cleavage occurs at blue arrows and forms blunt ends while forming sticky ends at gray arrows. *Bam*GI is taken as an example that represents endonuclease forming blunt ends. And *Bam*HI is taken as an example that represents endonuclease forming sticky ends. **D** The *mod* and *res* genes are arranged in series and transcribed by one promoter. **E** Type III R-M system recognizes and cleaves the exotic phage DNA in the form of a complex composed of two Mod subunits and one Res subunit. The complex function in the presence of two or more copies of recognition sequences arranged in an inverse orientation. Both head-to-head and end-to-end are valid. Mod subunit can function independently and methylate on a single strand. The blue arrows indicate the orientation of the recognition sites

The type I R-M system is a pentameric protein complex composed of three distinct subunits: HsdS (S), HsdM (M), and HsdR (R). These subunits are encoded by closely related *hsdS*, *hsdM* and *hsdR* genes. While HsdS and HsdM are essential for DNA methylation, the HsdR subunit is crucial for restriction activity (Cajthamlová *et al.* 2007). The type I methyltransferase (MTase) is functionally similar to an M_2_S_1_ complex (Taylor *et al.* 2012) and requires two HsdM subunits and one HsdS subunit for its activity. Each HsdS subunit contains two target recognition domains (TRDs) with distinct DNA recognition sequences. These TRDs are specifically involved in restriction and methylation activities in an antiparallel arrangement joined by two conserved regions (CRs), structurally acting as α-helices (Kennaway *et al.* 2012; Kim *et al.* 2005; Willemse and Schultsz 2016) (Fig. 1A). Furthermore, the trimeric M_2_S_1_ complex requires AdoMet as a cofactor to activate its MTase function (Taylor *et al.* 2012). For the restriction endonuclease (REase) activity, the M_2_S_1_ complex associates with two HsdR subunits to form the R_2_M_2_S_1_ complex (Fig. 1B). This assembly is required for DNA cleavage (Kennaway *et al.* 2012; Willemse and Schultsz 2016). The N-terminal endonuclease domain of the HsdR subunit is linked to a “motor” domain, similar to those found in other nucleic acid processing enzymes with translocase or helicase functions (Bialevich *et al.* 2017; Taylor *et al.* 2012). Furthermore, the restriction activity also requires Mg^2+^ as a cofactor and ATP hydrolysis allows the R_2_M_2_S_1_ complex to translocate DNA, facilitating cleavage at a distance from the recognition site (Bialevich *et al.* 2017; Dybvig *et al.* 1998; Obarska-Kosinska *et al.* 2008). Type I enzymes recognize asymmetric DNA sequences. For instance, the well-studied *Eco*R124I Type I R-M system recognizes the sequence GAAN_6_RTCG (where N represents any base) (Taylor *et al.* 2010). The spacer between the two recognition sequence elements can range from 4 to 8 nucleotides, as documented in REBASE (Roberts *et al.* 2023). Interestingly, DNA restriction occurs at least 1 kb away from the recognition site (Green and Sambrook 2021).

X

Not all Type I R-M systems in bacteria possess a complete set of *hsd* genes. According to analysis of 2145 bacterial and archaeal genomes in REBASE by Loenen *et al*. reveals that more than half contain at least one copy of the *hsdS*, *hsdM*, and *hsdR* genes. Approximately 39% of genomes lack *hsd* genes entirely, while the remaining genomes possess some but not all *hsd* genes (Loenen *et al.* 2014). Consequently, it is common for genomes to have one to three or more Type I R-M systems. For instance, some species of *Mycoplasma* have only one or two *hsdR* and *hsdM* genes but multiple *hsdS* genes (Loenen *et al.* 2014). However, due to the inversion within the *hsdS* gene, which results in non-functional *hsdR* and *hsdM* genes (Dybvig *et al.* 1998), this system becomes inoperative in *Mycoplasma pulmonis*. The diversity of type I R-M specificities often exceeds the number of *hsdS* genes present, which can be attributed to the different combinations of the individual TRDs of the subunits (Janscak and Bickle 1998). This combinatorial potential allows for a greater range of DNA recognition and modification capabilities.

The Type I R-M system is classified into five families based on complementation, hybridization, and sequence variations in their allelic genes (Roberts *et al.* 2012). These families are referred to as Type IA, IB, IC, ID, and IE. Notable examples include *Eco*BI (Kasarjian *et al.* 2003) and *Eco*KI (Roberts *et al.* 2012) in the Type IA family, *Eco*AI in Type IB (Kudryavtseva *et al.* 2023a), *Eco*R124I in Type IC family (Smith *et al.* 2001), *Sty*SBLI in Type ID family, and *Kpn*BI in Type IE (Cajthamlová *et al.* 2007). Among these subtypes, the Type IB systems are characterized by their smaller M subunits and larger S subunits compared to the other four subtypes (Roberts *et al.* 2012).

In addition to its primary role in protecting bacteria from phage infection, the type I R-M system has other important functions. In particular, it acts as a barrier to horizontal gene transfer (Waldron and Lindsay 2006), which helps prevent the spread and propagation of antibiotic resistance genes. This function is critical in mitigating the exacerbation of antibiotic resistance and its associated detrimental effects. Although the Type I R-M system has not been widely used as a tool in molecular biology, its three-dimensional structure and role as a molecular motor present promising avenues for further investigation. Exploring the relationship between its structural features and functional mechanisms could greatly enhance our knowledge of its function and contribute to the advancement of molecular biology.

### Type II R-M system

Type II R-M systems are the simplest and most common among the various R-M systems. These systems are typically integrated into host chromosomes; however, Type II REase genes are also occasionally found on mobile genetic elements such as plasmids (Khan *et al.* 2010). However, these genes are rarely observed in phages, whereas MTases are sometimes present in phages, probably as a self-protection mechanism (Gulati *et al.* 2023).

The Type II enzymes have been widely used as functional tools and have made great efforts in biomedical and molecular research (Green and Sambrook 2021). The system comprises two distinct components: a REase and an MTase. These enzymes operate independently but recognize and act upon the same DNA sequence, and their genes are typically linked on the same chromosome (Wilson 1991). In some subtypes, such as IIB, IIG and IIH, the REase and MTase are fused into a single composite gene, reflecting a unique structural arrangement within these systems (Roberts *et al.* 2003).

Type II R-M systems typically recognize palindromic DNA sequences of 4–8 bp in length (Orlowski and Bujnicki 2008). They exhibit two main cleavage patterns: one produces blunt ends by cleaving at the center of the symmetrical sequences (palindromes), while the other produces sticky ends by cleaving at corresponding positions on the two DNA strands (Green and Sambrook 2021) (Fig. 1C). Both cleavage types result in 5’-phosphates and 3’-hydroxyl groups (Brooks 1987; Green and Sambrook 2021; Roberts *et al.* 2003).

The activity of Type II REases requires Mg^2+^ as a cofactor (Kovall and Matthews 1998), although other metal ions such as Mn^2+^ and Zn^2+^ can substitute for Mg^2+^ in some cases (Gasiunas *et al.* 2008). Notably, Ca^2+^ ions sometimes inhibit cleavage by competing Mg^2+^ or Mn^2+^ binding sites (Chandrashekaran *et al.* 2004; Viadiu and Aggarwal 1998). It also increases the sequence specificity of several Type II REases (Saravanan *et al.* 2007). Methylation by Type II MTases requires AdoMet as a methyl group donor, similar to Type I MTases.

Hundreds of recognition and cleavage sequences have been identified for Type II R-M systems, allowing versatile characterization and manipulation of DNA by combining different restriction enzymes. These systems are further classified into families based on shared characteristics. Notably, subtypes of Type II enzymes are characterized and grouped rather than strictly categorized (Roberts *et al.* 2003). Below are the main subtypes of Type II R-M systems.

Type IIA. Recognizes asymmetric sequences and typically consists of one REase gene and two MTase genes (Roberts *et al.* 2003). Examples include *Fok*I and *Bau*I.

Type IIB. Cleaves on both sides of the recognized sequence, similar to Type I system but ATP independent (Bower *et al.* 2018). Examples include *Alo*I and *Eco*RIII.

Type IIC. Contains both cleavage and modification domains within a single polypeptide (Nakonieczna *et al.* 2009). Examples include *Gsu*I and *BcgI*.

Type IIE. Requires two copies of the recognition sequence, with one copy stimulating cleavage at the other (Sasnauskas *et al.* 2017). Examples include *Eco*57I and *Eco*RII.

Type IIF. Interacts with two copies of the recognition sequence and cleaves simultaneously (Khan *et al.* 2010). Examples include *Bbe*I and *Sfi*I.

Type IIG. Features a single polypeptide with combined catalytic centers for endonuclease and methyltransferase activity (Jurenaite-Urbanaviciene *et al.* 2007). These enzymes can be stimulated or inhibited by AdoMet (Roberts *et al.* 2003). Examples include *Acc*65V and *Bae*I.

Type IIH. Combines features of Type I MTases and Type II REases (McGeehan *et al.* 2005). Examples include *Bcg*I and *Sau*42I.

Type IIM. Recognizes specific methylated sequences for cleavage (Siwek *et al.* 2012). Examples include *Dpn*I and *Glu*I.

Type IIP. Recognizes a symmetrical sequence and cleaves at symmetrical sites within or adjacent to the site (Mucke *et al.* 2003). Examples include *Eco*RI and *Bam*HI.

Type IIS. Cleaves one or both DNA strands at a precise distance from the recognition site, making it a widely used enzyme in biotechnology (Kennedy *et al.* 2023). Examples include *Aar*I and *Bfu*I.

Type IIT. Consists of heterodimeric subunits (Smith *et al.* 2001). Examples include *Bpu*10I and *Bsi*I.

Due to the large number of Type II R-M REases, the concept of isoschizomers has been introduced. Isoschizomers are different REases that recognize the same DNA sequence regardless of their cleavage sites. The term is derived from the Greek, where "iso" means equal and "schizo" means split (Roberts 1976).

### Type III R-M system

The Type III R-M system exhibits some similarities to the Type I R-M system, particularly in its complex protein subunits composition, but is structurally and functionally simpler. The Type III R-M system was first described as a phage- or plasmid-encoded restriction system (Hadi *et al.* 1983), such as *Eco*P1I and *Eco*P15I that have been extensively studied (Janscak *et al.* 2001; Schwarz *et al.* 2013).

This system comprises two primary genes: *mod* and *res*, which encode the Mod and Res proteins, respectively. They are arranged in series and transcribed by a promoter in front of the *mod* gene (Iida *et al.* 1983) (Fig. 1D). The Mod subunit recognizes and methylates specific DNA sequences, while the Res subunit cleaves DNA. Together, these subunits form the Type III R-M system, which is typically a heterotrimer consisting of one Res subunit and two Mod subunits (Butterer *et al.* 2014). The Mod subunit mediates methylation, protecting host DNA by *N*^6^-methylating deoxyadenosine or *N*^4^-methyldeoxycytosine on a single strand of a non-palindromic sequence, can function independently (Murray *et al.* 2021). In contrast, restriction activity, depends on both the Mod and Res subunits (Fig. 1E).

During restriction, the Type III enzyme interacts with two or more copies of the non-palindromic recognition sequence arranged in an inverse orientation (Fig. 1E), which typically ranges from 5–6 bp in length (Sistla and Rao 2004). The cleavage site is located at a distance of ~25 bp on the 3’ side of the recognition sequence (Brooks 1987; Green and Sambrook 2021). Communication between two distal sites is essential prior to cleavage (Ahmad *et al.* 2018). The Res subunit has an ATP hydrolysis domain that drives protein translocation and ensures proper restriction activity, similar to the Type I R-M system (Murray *et al.* 2021; Roberts *et al.* 2003). AdoMet acts as an allosteric activator for restriction (Brooks 1987). It has also been shown that the *Eco*P15I REase can bind substrate DNA in the absence of AdoMet without any impact on the rate or yield of the process, as *Eco*P15I is inherently bound to AdoMet, eliminating the need for an additional cofactor (Bist *et al.* 2001). Interestingly, the presence of exogenous AdoMet can reduce the efficiency of R.*Eco*P15I (Restriction enzyme of *Eco*P15I) by means of suppressing cleavage within linear form of DNA (Raghavendra and Rao 2005).

The Type III R-M system is reminiscent of the Type I R-M system as Type III R-M system from clinical *Staphylococcus* aureus can show as the barrier of horizontal gene transfer (Corvaglia *et al.* 2010), which is similar to Type I R-M system. And both systems function as multisubunit complexes. However, the distinct arrangement and interaction of functional subunits within the Type III system confers unique capabilities.

### Type IV R-M system

**Figure 2:**
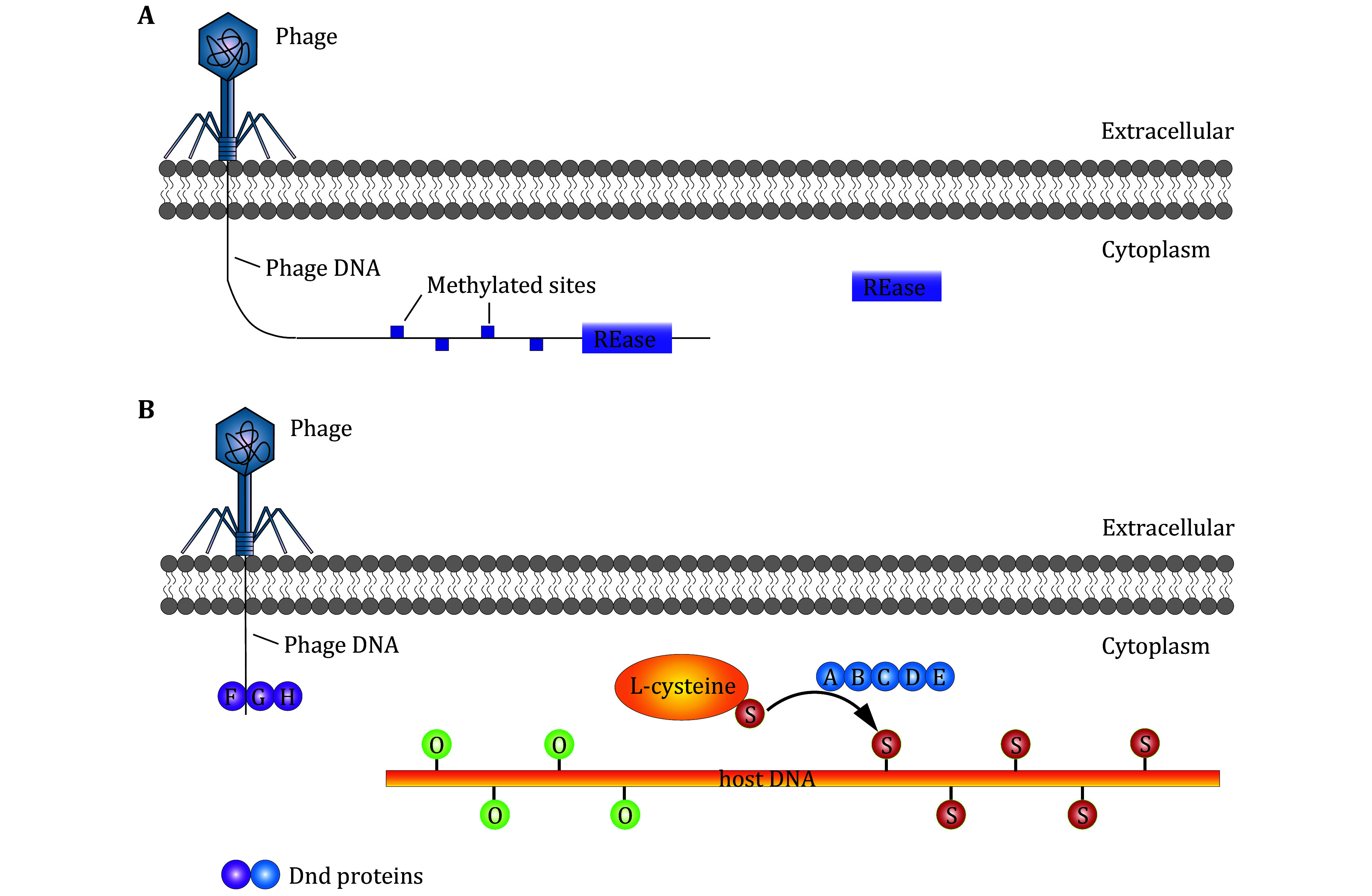
Mechanisms of Type IV and PT R-M Systems to cleave exotic phage DNA. **A** REase in Type IV R-M system cleaves the sequences that are modified. **B** Dnd A-E protein modify DNA backbones by facilitating sulfur transfer from L-cysteine to replace the oxygen atom on it. Dnd F-H protein recognizes and cleaves unmodified exotic phage DNA

The type IV R-M system is fundamentally different from types I, II, and III in that it cleaves only modified DNA (Fig. 2A). The endonuclease of this system recognizes and acts on methylated, hydroxymethylated and glucosyl-hydroxymethylated bases. Its activity is GTP dependent. The role of DNA modification in restriction was first observed in phage T4 populations, where 5-hydroxymethyl cytosine (hm^5^C) modifications were detected (Luria and Human 1952). Later, host-derived glucosylation, facilitated by phage-encoded glucosyltransferase, was discovered (Georgopoulos and Revel 1971; Hattman and Fukasawa 1963). In the 1980s, the identification of 5-methylcytosine (m^5^C) led to the mapping of the *mcrA* and *mcrB* genes associated with Type IV activity (Raleigh and Wilson 1986). Additionally, the discovery of hm^5^C in higher eukaryotic DNA spurred further interest in these systems (Kriaucionis and Heintz 2009; Penn 1976; Penn *et al.* 1972; Tahiliani *et al.* 2009).

The McrBC endonuclease is the well-known Type IV system, consisting of three proteins encoded by the *mcrB* and *mcrC* genes. The *mcrB* gene produces McrBL, a full-length protein, and McrBS, a truncated variant of McrBL produced by internal translation. McrC is essential for DNA cleavage, which requires a precise ratio of McrBL to McrC, with McrBS acting as a regulator (Stewart *et al.* 2000). Besides, McrB and McrC have other functions. McrB protein hydrolyzes GTP, a process that can be simulated by McrC protein by means of binding onto the single active site of McrB. The asymmetric structure of the McrB protein directs the process and thus equips with the optimal catalytic function (Niu *et al.* 2020). This process provides energy for the DNA translocation between distant recognition elements as the cleavage needs at least two recognition sites which are separated by 40 to 3000 bp (Panne *et al.* 1999).

The *Eco*57I enzyme is another example of Type IV enzyme. It has both restriction and modification capabilities within a single polypeptide, making it a bifunctional enzyme (Jurenaite-Urbanaviciene *et al.* 2001). *Eco*57I methylates one strand of its asymmetric recognition sequence. A separate *Eco*57I methyltransferase modifies both strands. The amino acid sequence of R.*Eco*57I contains both the catalytic DNA cleavage motif and the Mg^2+^ binding motif, and its restriction activity is simulated by AdoMet (Jurenaite-Urbanaviciene *et al.* 2001). *Eco*57I is thought to have evolved from the fusion of the Mod and Res subunits of the Type III R-M enzyme, sharing similarities in cleavage mechanism, metal ion dependencies, and AdoMet simulation, while operating in the absence of ATP (Janulaitis *et al.* 1992).

Type IV enzymes have great potential in genomic studies. They facilitate clone library enrichment by targeting highly methylated, transcriptionally silenced DNA for remodeling and reactivation (Palmer *et al.* 2003). This capability provides powerful tools for eukaryotic genomic studies, revealing insights that were previously inaccessible.

### PT R-M system

PT R-M system is a unique one that differs from the four classical R-M systems. Unlike conventional systems that modify nucleobases by methylation, PT enzymes target the sugar-phosphate backbone of DNA. This modification involves the substitution of a sulfur atom for a non-bridging oxygen atom in the phosphodiester backbone, a process known as phosphorothioation (Wang *et al.* 2007). The Dnd PT system was the first PT R-M system identified. The modification process is mediated by five key proteins: DndA, DndB, DndC, DndD and DndE, which facilitate the transfer of sulfur from L-cysteine, enabling the DNA backbone modification in a manner that is both sequence-specific and stereospecific (Wang *et al.* 2007). The restriction component of the Dnd PT system comprises the DndF, DndG, and DndH proteins, which recognize and cleave unmodified DNA in a non-sequence-specific manner (Wu *et al.* 2022). Through the cooperative action of the modification (DndA-E) and restriction (DndF-H) proteins, the Dnd PT system protects host DNA while targeting foreign DNA for cleavage (Wang *et al.* 2019; Xiong *et al.* 2020) (Fig. 2B).

A second PT R-M system, the SspABCD-SspE PT system, was found in 2020. This system, found in bacteria such as *Vibrio cyclitrophicus*, *Escherichia coli*, and *Streptomyces yokosukanensis*, targets single-stranded DNA (ssDNA). Unlike the Dnd PT system, which operates on double-stranded DNA (dsDNA), the SspABCD-SspE system functions through DNA nicking. And they protect distinct consensus sequences (Xiong *et al.* 2020). The consensus modification sequence in the Dnd system is 5’-GAAC-3’/5’-GTTC-3’. In contrast, the Ssp system targets the motif 5’-C_PS_CA-3’ (where ‘PS’ indicates a phosphate-sulfur bond). The Ssp proteins show functional parallels to their Dnd counterparts. For example, SspA and SspD function analogously to DndA and DndC, serving as cysteine desulfurase and pyrophosphatase, respectively (You *et al.* 2007). SspC possesses ATPase activity that drives the modification process similarly to DndD. Notably, the *sspB* gene encodes a protein that shares no homology with any Dnd proteins, highlighting a unique function within the ssDNA PT modification system. The SspB protein acts as a nickase, cleaving ssDNA and creating nicks on the strand. For example, it introduces two or more nicks on dsDNA, such as pUC19, to break it (Xiong *et al.* 2020).

In addition to its extensive role in bacterial defense, the PT R-M system has been implicated in several critical cellular functions. In particular, it contributes to the maintenance of the cellular redox state and may also exert effects on epigenetic regulation (Wang *et al.* 2019; Xie *et al.* 2012). Recent studies suggest that the PT R-M system may help reduce the abundance of antimicrobial resistance genes in the genome. Although the evidence linking PT systems and antimicrobial resistance is not yet definitive, these findings open up potential therapeutic avenues to address antimicrobial resistance (Xu *et al.* 2023).

## PHAGE COUNTERMEASURES

The evolutionary arms race between bacteria and bacteriophages has driven the development of intricate survival mechanisms on both sides. Bacteria employ R-M systems as a key defense, while phages have evolved countermeasures to evade or neutralize these bacterial defenses. This ongoing conflict highlights the adaptive complexity of host-pathogen interactions.

### DNA modification

**Figure 3:**
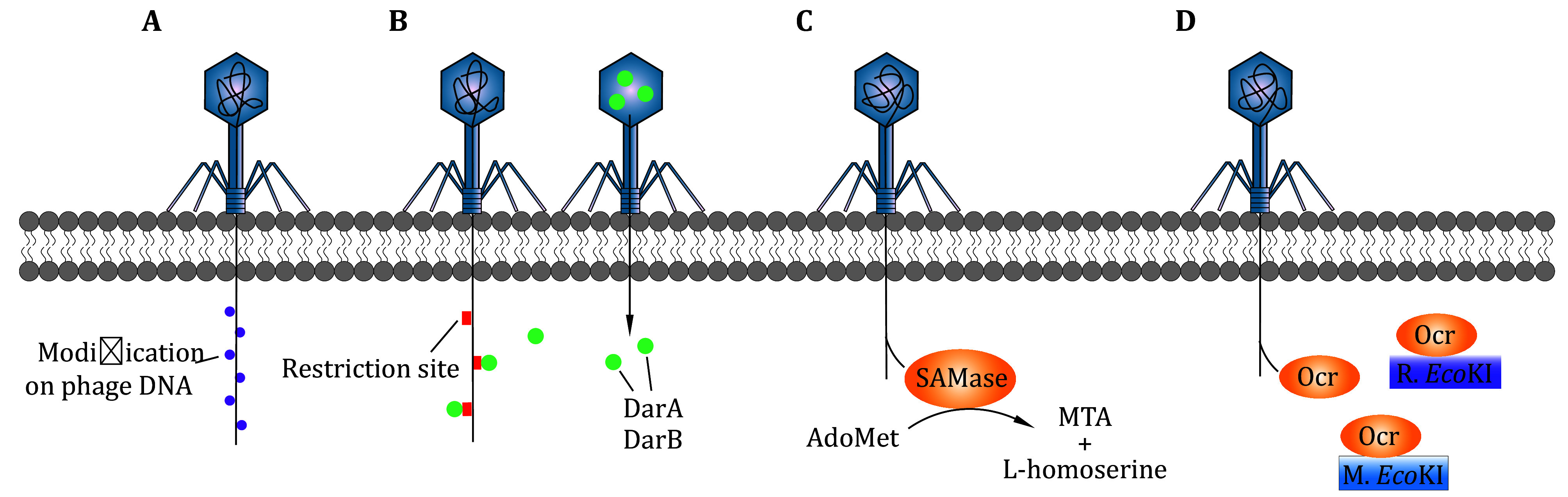
Different strategies used by phage to evade bacterial R-M systems.** A** Phage modifies its own DNA to escape host restriction. The purple balls indicate modification on phage DNA, which can be methyl or glucosyl. **B** Phage P1 co-injects DarA and DarB proteins encoded by itself. The two proteins can shield the restriction sites on their DNA. **C** Phage T3 expresses SAMase to degrade AdoMet. **D** Phage T7 encodes Ocr protein that can competitively bind with R.*Eco*KI and M.*Eco*KI

To avoid cleavage by restriction enzymes targeting unmodified DNA, some phages preemptively modify their own DNA prior to being recognized by REase (Fig. 3A). A DNA MTase, which has the same sequence specificity as M.*Bsu*RI (Modification enzyme of *Bsu*RI), can be encoded by prophage and methylate phage DNA. Thus, the phage DNA can escape recognition by R.*Bsu*RI (Noyer-Weidner *et al.* 1981; Trautner *et al.* 1980). For instance, phage T4 encodes a glucosyltransferase that modifies its DNA during replication in *Escherichia coli*. Specifically, 5-hydroxymethyl dCTP is incorporated into newly synthesized DNA in place of dCTP, and the 5-hydroxymethylcytosines are further glucosylated by the phage-encoded glucosyltransferase, which confers phage T4 resistance to Type I-III R-M systems (Wang *et al.* 2023). Phages can also recruit host MTases to methylate their genomes. For example, the Ral protein in phage λ enhances the modification activity of host MTases (Zabeau *et al.* 1980). Thus, the phage λ genome can be modified and then escape from restriction.

### Decrease of restriction sites

Phages may reduce or shield the number of restriction sites targeted by bacterial R-M systems. Phages reduce susceptible sequences through point mutations. For example, the T4 phage replaces normal cytosine with hm^5^C to avoid cleavage (O'Farrell *et al.* 1980). In addition to sequence modifications, phages utilize other strategies to shield their restriction sites by binding with accessory proteins encoded by phage DNA. For example, phage P1 encodes the DarA and DarB proteins, which are co-injected with phage DNA into host cells. These proteins occlude the restriction sites on the phage P1 DNA and avoid being destroyed by the R-M system (Iida *et al.* 1987) (Fig. 3B). However, restriction avoidance is not a universal strategy. It is more prevalent in DNA viruses, particularly in non-temperate phages targeting Type II and Type IV R-M systems (Rusinov *et al.* 2018). And it’s competitive with other anti-restriction mechanisms. Take *Myoviridae* family as an example, they rely on other mechanisms like encoding DNA hydroxymethylases, reducing the need for extensive restriction site avoidance. (Rusinov *et al.* 2018).

### Cofactors degradation

To function effectively, R-M systems require several cofactors, including AdoMet. AdoMet is a vital cofactor for both Type I and III R-M systems. Specifically, Type I-III R-M systems use AdoMet as a methyl donor, while Type III restriction is AdoMet allosteric activated. Phage T3 has evolved a strategy to counteract these R-M systems by expressing an AdoMet hydrolase enzyme (also known as SAMase) that functions by degrading AdoMet into 5’-methylthioadenosine (MTA) and L-homoserine (Gefter *et al.* 1966), thereby neutralizing the activity of both Type I and III R-M systems (Studier and Movva 1976) (Fig. 3C). Additionally, AdoMet hydrolase can interfere with the host’s methionine *S*-adenosyltransferase, the enzyme responsible for producing AdoMet, further inhibiting R-M system functionality (Simon-Baram *et al.* 2021). It is important to note that this AdoMet hydrolase cannot inhibit the R-M enzymes that are already bound to AdoMet (Tock and Dryden 2005).

### Proteins inhibit restriction

Some phages express anti-restriction proteins to evade host R-M systems. One such protein is Ocr (overcome classical restriction), encoded by gene *0.3* of bacteriophage T7 (Studier 1975). Ocr mimics the size and shape of B-form DNA and can bind with the Type I enzyme *Eco*KI with high affinity (Walkinshaw *et al.* 2002). This binding effectively inhibits both the restriction and modification activities of *Eco*KI, thereby facilitating phage survival (Fig. 3D). Additionally, phage T3 also encodes the Ocr protein. And it can inhibit the Type III R-M system while inhibiting the Type I (Krüger *et al.* 1982).

Another anti-restriction protein, ArdA (alleviation of restriction of DNA), encoded by the *ard* gene family, exhibits a similar function to Ocr by mimicking DNA and inhibiting the Type I R-M system. However, whether ArdA inhibits restriction or methylation activity depends on its interactions with the Type I R-M enzyme (Nekrasov *et al.* 2007), which is different from those of the Ocr protein. For instance, ArdA proteins encoded by plasmids R16 and R64 specifically inhibit the restriction activity of Type I system (Balabanov *et al.* 2012; Thomas *et al.* 2003), whereas ArdA encoded by plasmids such as pKM101 and ColIb-P9 can inhibit both restriction and modification functions (Belogurov *et al.* 1993; Zavilgelsky *et al.* 2008). Additionally, the ArdB protein inhibits the Type I R-M system by interacting with the R subunit of the enzyme complex (Kudryavtseva *et al.* 2020). Unlike ArdA and Ocr, ArdB does not utilize a DNA mimicry mechanism, although the precise details of its inhibitory action remain to be fully elucidated (Kudryavtseva *et al.* 2023b).

## DISCUSSION

The R-M system is a major defense system that is divided into five types based on their specific modes of action. The mechanisms of the different types suggest that the R-M system is a product of long-term evolution. For instance, the Type IIS R-M enzyme, *Eco*57I, is very similar to Type III enzymes with only minor differences, which serves as a good example of the evolution of the R-M system. The phage counterattack has diversified, corresponding to the diversity of the R-M system. This suggests that the R-M system and phages have co-evolved during a long-term arms race. Although the R-M system has been well studied, there are still many unknowns about this bacterial defense mechanism and the detailed mechanisms of phage countermeasures. This may remind us that we still have a lot to learn about R-M systems in order to explore the molecular mechanisms. Additionally, the co-evolution of R-M systems and phage countermeasures could be an excellent aspect. At the same time, as the problem of antibiotic resistance is becoming epidemic, people need new therapies against pathogens. A deeper understanding of the mechanism by which the R-M system resists phages could help us overcome the major limitation of phage therapy. The regulation of horizontal gene transfer is also a barrier to the spread of antibiotic resistance genes (Fernandez-Lopez and de la Cruz 2014). Other studies have shown that the presence of the PT R-M system reduces the frequency of antibiotic resistance genes in the genome (Xu *et al.* 2023). However, the negative correlation between the two remains to be proven. In addition, the Type IV R-M system has been shown to provide a way to remodel silenced genes. This will enrich the genome and help us to improve our understanding of these genes.

Overall, significant progress has been made in unraveling the complexity of the R-M system. Future research will not only deepen our understanding of its molecular mechanisms, but may also provide new therapeutic strategies against bacterial pathogens. Moreover, the study of the intricate structure-function relationship of the R-M system may provide innovative tools for genomic analysis and investigation, highlighting the broader implications and significance of this research.

## Conflict of interest

Haoyang Kang, Ang Gao and Yalan Zhu declare that they have no conflict of interest.
